# Beyond deficiency prevention: meteorological determinants and nonlinear associations of maternal vitamins D, A, and E with perinatal outcomes in 10,824 Chinese pregnancies

**DOI:** 10.3389/fnut.2026.1737197

**Published:** 2026-02-25

**Authors:** Changzhen Li, Shiyong Deng, Ping Zhou, Jingjing Rao, Yun Xiang, Lei Xi, Xiaomei Wang

**Affiliations:** 1 Department of Laboratory Medicine, Wuhan Children’s Hospital (Wuhan Maternal and Child Healthcare Hospital), Tongji Medical College, Huazhong University of Science & Technology, Wuhan, China; 2 Department of Gynecology, Wuhan Children’s Hospital (Wuhan Maternal and Child Healthcare Hospital), Tongji Medical College, Huazhong University of Science & Technology, Wuhan, China

**Keywords:** environmental determinants, fat-soluble vitamins, maternal nutrition, nonlinear associations, outcomes, perinatal, subtropical populations

## Abstract

**Objective:**

To examine meteorological factors associated with maternal vitamins D, A, and E and their associations with delivery and neonatal outcomes in central China.

**Methods:**

In this retrospective cross-sectional analysis of 10,824 third-trimester women who delivered at 37 weeks or later in Wuhan (2020–2023), serum vitamin levels were measured by high-performance liquid chromatography. Daily temperature, humidity, precipitation, and wind speed were matched to individual blood collection dates. Multivariable logistic regression and restricted cubic spline models were used to evaluate associations with fetal distress, meconium-stained amniotic fluid, premature rupture of membranes (PROM), low birth weight, and macrosomia.

**Results:**

Vitamin D deficiency (30.2%), vitamin A deficiency (5.5%), and vitamin E excess (41.8%) were common. Each 1 °C increase in ambient temperature was associated with lower odds of vitamin D deficiency (aOR 0.970, *p* < 0.001) and vitamin A deficiency (aOR 0.976, *p* = 0.008), with significant temperature–humidity and temperature–wind interactions (*p* < 0.001). Vitamin A deficiency was associated with higher odds of PROM (aOR 1.76, 95% CI 1.44–2.14), whereas low or moderate vitamin D and A levels were associated with lower odds of fetal distress and meconium staining (aOR approximately 0.85, *p* < 0.05). Vitamin A deficiency was associated with markedly higher odds of macrosomia (aOR 3.14), and vitamin E excess was associated with a 60% increase in odds (aOR 1.60). Restricted cubic spline models revealed U-shaped associations between vitamin A and low birth weight, and between vitamin D and macrosomia.

**Conclusion:**

Ambient temperature emerged as the primary meteorological factor associated with maternal vitamin status. Both deficiency and excess were associated with adverse delivery and neonatal outcomes, supporting the need for population-specific optimization beyond deficiency prevention.

## Introduction

Maternal vitamin D deficiency affects approximately 54% of pregnant women globally (1), with prevalence reaching 74–84% in Chinese populations (2, 3). However, these rates remain inadequately characterized in subtropical Asian regions. Vitamin D deficiency is a major global public health challenge associated with adverse maternal and child health outcomes across diverse populations (4, 5). Emerging evidence challenges the traditional focus on deficiency prevention, as maternal fat-soluble vitamins show U-shaped associations with perinatal outcomes (6, 7), where both deficiency and excess pose risks. For vitamin D, optimal birth outcomes are observed at intermediate 25(OH)D levels (6); for vitamin A, both deficiency and excessive intake are linked to adverse fetal outcomes (8); and elevated maternal vitamin E concentrations are associated with macrosomia in Chinese cohorts (7). These findings necessitate a paradigm shift toward population-specific optimization rather than indiscriminate supplementation (9, 10).

While demographic and nutritional determinants have been extensively characterized (5), environmental modifiers, particularly meteorological factors, remain inadequately understood for vitamins A and E. Ambient temperature has emerged as the strongest environmental predictor of vitamin D levels (11, 12), with each 1 °C increase associated with a 3–4% reduction in deficiency risk (11). The mechanistic basis involves UVB-mediated cutaneous synthesis of vitamin D₃, which is highly dependent on solar zenith angle and atmospheric conditions (13, 14). Recent Chinese cohorts confirm marked seasonal variation, with summer 25(OH)D concentrations exceeding winter values by 20–30% (3, 15). However, the influences of humidity, precipitation, and wind speed, especially their interactive effects with temperature, remain largely unexplored. This knowledge gap is particularly evident for vitamins A and E, where meteorological associations have not been systematically investigated despite plausible links through seasonal dietary patterns and temperature-related metabolic changes.

Accumulating evidence reveals nonlinear dose–response relationships, highlighting the need to identify optimal ranges beyond simple deficiency thresholds. For vitamin D, observational studies document U-shaped associations with small-for-gestational-age risk, with nadirs at 25–40 ng/mL (6, 16, 17). Several cohorts report similar patterns for birth weight, where both low (<20 ng/mL) and high (>40 ng/mL) maternal 25(OH)D levels are associated with suboptimal outcomes (18). Vitamin A demonstrates biphasic toxicity: deficiency (<200 ng/mL) correlates with maternal anemia and infections (8), while excessive intake (>10,000 IU/day) increases teratogenic risk fourfold (19). For vitamin E, a large Chinese cohort (n = 8,554) reported that concentrations exceeding 20,000 ng/mL were associated with increased risk of macrosomia (aOR = 1.60) (7). Micronutrient optimization in pregnancy requires context-specific approaches given population heterogeneity in nutritional status and environmental exposures (20, 21). Critically, these nonlinear patterns appear population-specific (6, 7, 18), underscoring the need for regional calibration rather than universal application of Western reference ranges.

Geographic representation in maternal vitamin research remains skewed toward high-latitude temperate (>40°N) and coastal subtropical regions (1, 22), leaving inland subtropical populations underrepresented. Wuhan (30°N, central China) serves as an ideal natural laboratory: its humid subtropical climate features extreme seasonal meteorological variation, allowing examination of environmental influences within a single population (11). Additionally, rapid nutrition transition (23) and increasing maternal obesity prevalence [37.5% in our cohort with BMI > 28 kg/m^2^, consistent with national trends (24)] may result in distinctive vitamin profiles in contemporary Chinese urban populations. Despite these unique characteristics, no previous studies have simultaneously assessed vitamins D, A, and E with comprehensive meteorological integration in inland subtropical China.

Three critical knowledge gaps hinder the optimization of maternal vitamin supplementation in Asian populations. First, environmental determinants beyond latitude, particularly the interactive effects of temperature, humidity, and wind speed, remain uncharacterized for vitamins A and E, despite well-established influences on vitamin D. Second, dose–response relationships may exhibit population-specific thresholds, yet Western-derived reference ranges have not been validated in Chinese cohorts using continuous modeling approaches. Third, most studies focus on single micronutrients, limiting the assessment of potential synergistic or antagonistic interactions among vitamins D, A, and E across multiple perinatal outcomes. However, whether these associations reflect causal effects or late-pregnancy correlates remains uncertain, highlighting the need for prospective studies with serial measurements.

To address these gaps, we conducted a retrospective cohort study of 10,824 third-trimester pregnant women in Wuhan, China (2020–2023), integrating HPLC-measured serum vitamins, daily meteorological data, and comprehensive clinical records. Our objectives were to: (1) characterize the distributions of vitamins D, A, and E and their meteorological determinants, with an emphasis on interactive effects; (2) quantify associations with five perinatal outcomes using sequential adjustment models; and (3) evaluate dose–response patterns through restricted cubic spline regression. By simultaneously assessing three vitamins with environmental integration, this study provides a framework for optimizing maternal micronutrient status in subtropical Asian populations.

## Materials and methods

### Study design and population

This retrospective cross-sectional study analyzed data from 10,824 pregnant women who received prenatal care and delivered at Wuhan Maternal and Child Healthcare Hospital, Wuhan, China, between 2020 and 2023. Although participants were drawn from a delivery cohort, vitamin concentrations were measured only once in late pregnancy; therefore, analyses should be interpreted as cross-sectional with respect to vitamin exposure. Inclusion criteria were: (1) singleton pregnancy; (2) gestational age of at least 37 weeks at delivery; (3) maternal age of at least 18 years; (4) availability of serum vitamin measurements obtained at 37–40 weeks of gestation; (5) complete medical records including demographic characteristics, anthropometric data, meteorological variables, and pregnancy outcomes; and (6) residence in the study region for at least 6 months before delivery. Exclusion criteria were: (1) multiple gestations; (2) preterm delivery (less than 37 weeks); (3) pre-existing chronic conditions known to affect vitamin metabolism (e.g., chronic liver or kidney disease, malabsorption syndromes, parathyroid disorders); (4) incomplete or missing data on key variables; and (5) pregnancies complicated by fetal anomalies or chromosomal abnormalities.

### Demographic and clinical variables

Maternal demographic and clinical data were extracted from electronic medical records by trained research personnel. Variables included date of blood collection, maternal age at delivery (years), age at menarche, age at marriage, gravidity, parity, gestational age at blood sampling (weeks), and body mass index (BMI, kg/m^2^). BMI was calculated from weight and height measurements obtained at the last prenatal visit before delivery and categorized into tertiles based on the study population distribution, as in previous studies (25): T1 (lowest tertile, 17.6–25.4 kg/m^2^), T2 (middle tertile, 25.5–28.0 kg/m^2^), and T3 (highest tertile, 28.1–59.5 kg/m^2^). Maternal age was categorized as <25, 25–29, 30–34, and ≥35 years. Residential location was classified as urban core or outer district based on the administrative divisions of Wuhan city.

### Meteorological data

Daily meteorological data were obtained from the China Meteorological Data Network. For each participant, the following meteorological variables were recorded on the day of blood collection: average temperature (°C), relative humidity (%), precipitation (mm), and wind speed (km/h). These measurements represented the daily average values for the 24-h period of blood sampling. The season of blood collection was classified as spring (March–May), summer (June–August), autumn (September–November), or winter (December–February) based on the date of blood sampling. For categorical analyses, meteorological variables were stratified into tertiles based on their observed distributions during the study period (2020–2023): T1 (lowest tertile), T2 (middle tertile), and T3 (highest tertile), except for precipitation which was dichotomized at the median (T1: low, T2: medium) due to its highly skewed distribution with a large proportion of zero values on non-rainy days. Participants were then assigned to tertile groups according to the meteorological conditions on their respective blood collection dates. To ensure temporal alignment between environmental exposure and biochemical assessment, meteorological variables on the exact date of blood sampling were used as exposure indicators, consistent with previous vitamin–climate studies (11–13).

### Specimen collection and detection

Fasting venous blood samples were collected in the morning at 37–40 weeks of gestation during routine prenatal visits. Blood samples were allowed to clot at room temperature for 30 min, then centrifuged at 3,000 rpm for 10 min. Serum was separated immediately and stored at −80 °C until analysis. Serum concentrations of 25-hydroxyvitamin D [25(OH)D], retinol (vitamin A), and *α*-tocopherol (vitamin E) were measured using high-performance liquid chromatography (HPLC). Vitamin D status was classified as deficient (<20 ng/mL), insufficient (20–30 ng/mL), or sufficient (≥30 ng/mL), in accordance with widely used clinical and epidemiological definitions for pregnant populations, including studies conducted in Chinese pregnant cohorts (26, 27). Vitamin A status was categorized as deficient (<200 ng/mL), insufficient (200–300 ng/mL), or sufficient (≥300 ng/mL), based on thresholds commonly applied in studies of maternal vitamin A status during pregnancy, including Asian and Chinese pregnant populations (8, 28). Vitamin E status was classified as deficient (<5,000 ng/mL), sufficient (5,000–20,000 ng/mL), or excessive (≥20,000 ng/mL), following reference ranges used in large-scale studies of pregnant women, including studies from East Asian populations (7). All assays were performed in the certified clinical laboratory of Wuhan Maternal and Child Healthcare Hospital, which participates in external quality assurance programs. Daily calibration was performed using certified reference materials, and internal quality control samples were analyzed with each batch. Intra-assay and inter-assay coefficients of variation were <5% for all vitamin measurements.

### Delivery and neonatal outcomes

Five delivery and neonatal outcomes were analyzed, all objectively extracted from electronic medical records at delivery. Delivery outcomes included fetal distress, meconium-stained amniotic fluid, and premature rupture of membranes (PROM). Neonatal outcomes included low birth weight (LBW, <2,500 g) and macrosomia (≥4,000 g). Each outcome was coded as a binary variable (1 = presence, 0 = absence).

### Statistical analysis

Statistical analyses were performed using R software (version 4.3.0; R Foundation for Statistical Computing, Vienna, Austria). Continuous variables are presented as mean ± standard deviation (SD) or median with interquartile range (IQR). Categorical variables are presented as frequencies and percentages. Distributions of serum vitamin levels were examined overall and across demographic, seasonal, and meteorological strata. For overall group comparisons, normality was assessed using the Shapiro–Wilk test. Between-group differences were evaluated using ANOVA for normally distributed variables or the Kruskal-Wallis test for non-normally distributed variables. Categorical variables were compared using the χ^2^ test. For specific pairwise comparisons in stratified analyses, all possible pairwise combinations were tested using Wilcoxon rank-sum tests, with *p*-values adjusted using Bonferroni correction to control the family-wise error rate. Spearman correlation coefficients quantified relationships between meteorological parameters and vitamin concentrations, and scatter plots with locally estimated scatterplot smoothing (LOESS) illustrated potential non-linear trends. Univariate and multivariable logistic regression models were used to identify predictors of vitamin deficiency and excess. To evaluate associations between maternal vitamin status and delivery or neonatal outcomes, three sequential logistic models were constructed: Model 1 (crude), Model 2 (adjusted for age and BMI), and Model 3 (fully adjusted for age, BMI, season, district, and gestational age). Information on gestational diabetes, hypertensive disorders, dietary intake, and supplement use was not available and therefore could not be included as covariates.

Potential non-linear dose–response associations between continuous vitamin concentrations and outcomes were further examined using restricted cubic spline (RCS) regression models implemented via the rms package in R. Four knots were specified at the 5th, 35th, 65th, and 95th percentiles of each vitamin distribution, following established recommendations by Harrell for large epidemiological datasets to balance model flexibility and overfitting risk. This knot placement strategy has been widely adopted in nutritional and environmental epidemiology studies (29, 30). RCS generate orthogonalized spline basis functions, which substantially reduce multicollinearity among spline terms. In addition, each RCS model included only one continuous vitamin exposure at a time, thereby minimizing potential collinearity between exposure terms. All models were adjusted for the same covariates as in categorical analyses (Model 3): maternal age group, BMI tertile, season of blood collection, residential district, and gestational age at sampling. Overall associations and departures from linearity were evaluated using likelihood ratio tests. We report two *p*-values for each vitamin-outcome pair: (1) *P_overall_*, testing the global association by comparing the spline model to a null model, and (2) *P_nonlinear_*, testing departure from linearity by comparing the spline model to a simple linear term. Adjusted odds ratios (aOR) with 95% confidence intervals (CI) were plotted across the observed exposure range, with all predictions referenced to the population median concentration (OR = 1.0 at median). Optimal concentration ranges defined as values where the upper bound of the 95% confidence interval remained ≤1.0, indicating statistically significant protective association (31). All ranges were restricted to the central 90% of observed vitamin distributions (5th to 95th percentile) to prevent extrapolation beyond regions with reliable data (32). For vitamin-outcome pairs where no concentration range met the 95% CI criterion, we do not designate an ‘optimal range’ due to lack of statistical evidence for risk reduction (95% CI crossing 1.0 throughout the observable distribution).

To examine effect modification by meteorological factors, interaction analyses were conducted using multivariable regression models. All continuous meteorological variables (temperature, humidity, precipitation, and wind speed) were standardized using z-score transformation (mean = 0, standard deviation = 1) prior to model fitting. Interaction terms were constructed as the product of standardized variables (e.g., temperature × humidity), allowing direct comparison of interaction coefficients across different meteorological factors on a standardized scale. Because variables were standardized, interaction coefficients represent relative effect modification rather than absolute changes in vitamin concentrations. All interaction models were adjusted for maternal age, BMI, gestational age, gravidity, parity, season, and residential district. Statistical significance was defined as a two-sided *p* < 0.05.

## Results

### Cohort characteristics and vitamin status distribution

A total of 10,824 pregnant women were included, with a mean maternal age of 31.2 ± 3.6 years (Table 1). Most participants were aged 25–34 years, nulliparous (68.1%), and resided in urban core districts (55.3%). The mean body mass index was 27.1 ± 3.3 kg/m^2^, distributed across tertiles (T1: 17.6–25.4 kg/m^2^; T2: 25.5–28.0 kg/m^2^; T3: 28.1–59.5 kg/m^2^). Blood samples were collected at a mean gestational age of 39.1 ± 0.9 weeks, with a relatively balanced seasonal distribution. Maternal vitamin status showed substantial heterogeneity and a high prevalence of abnormalities. The mean serum 25(OH)D concentration was 28.0 ± 12.6 ng/mL, with 30.2% of women classified as vitamin D deficient and 29.5% as insufficient. Serum vitamin A concentrations averaged 365.8 ± 114.7 ng/mL, with approximately one-quarter of participants exhibiting insufficiency, while overt deficiency was uncommon. In contrast, vitamin E excess was strikingly prevalent, with 41.8% of women having serum *α*-tocopherol concentrations ≥20,000 ng/mL and no cases of deficiency observed. Delivery and neonatal outcomes were available for all participants. The most frequent delivery complications were premature rupture of membranes (17.8%), fetal distress (16.5%), and meconium-stained amniotic fluid (14.6%). Neonatal outcomes included macrosomia in 3.6% and low birth weight in 1.9% of births, with a mean birth weight of 3,283 ± 582 g.

**Table 1:** Baseline characteristics of the study population.

Characteristic	Value (*N* = 10,824)
Maternal age, years	31.2 ± 3.6
<25	338 (3.1)
25–29	3,916 (36.2)
30–34	4,960 (45.8)
≥35	1,610 (14.9)
Body mass index, kg/m^2^	27.1 ± 3.3
T1 (lowest tertile)	17.6–25.4
T2 (middle tertile)	25.4–28.0
T3 (highest tertile)	28.1–59.5
Gestational age, weeks	39.1 ± 0.9
Nulliparous	7,374 (68.1)
Urban residence	5,989 (55.3)
Season of blood collection	
Spring	2,565 (23.7)
Summer	3,305 (30.5)
Autumn	2,734 (25.3)
Winter	2,220 (20.5)
Serum vitamin D, ng/mL	28.0 ± 12.6
Median (IQR)	26.5 (18.4–36.2)
Deficiency (<20 ng/mL)	3,264 (30.2)
Insufficiency (20–30 ng/mL)	3,198 (29.5)
Sufficiency (≥30 ng/mL)	4,362 (40.3)
Serum vitamin A, ng/mL	365.8 ± 114.7
Median (IQR)	355.4 (285.3–436.0)
Deficiency (<200 ng/mL)	599 (5.5)
Insufficiency (200–300 ng/mL)	2,611 (24.1)
Sufficiency (≥300 ng/mL)	7,614 (70.3)
Serum vitamin E, ng/mL	19,729 ± 5,452
Median (IQR)	18,987 (15956–22,606)
Deficiency (<5,000 ng/mL)	0 (0.0)
Sufficiency (5000–20,000 ng/mL)	6,297 (58.2)
Excess (≥20,000 ng/mL)	4,527 (41.8)
Delivery outcomes	
Fetal distress	1785 (16.5)
Meconium-stained amniotic fluid	1,576 (14.6)
Premature rupture of membranes	1926 (17.8)
Neonatal outcomes	
Low birth weight (<2,500 g)	202 (1.9)
Macrosomia (≥4,000 g)	391 (3.6)
Birth weight, g	3,283 ± 582

Data are mean ± s.d. for continuous variables and *n* (%) for categorical variables. Body mass index was measured at the last prenatal visit before delivery and was categorized into tertiles based on its distribution in the study population: T1 (lowest tertile, 17.6–25.4 kg/m^2^), T2 (middle tertile, 25.5–28.0 kg/m^2^), and T3 (highest tertile, 28.1–59.5 kg/m^2^). IQR, interquartile range.

### Vitamin status stratified by demographic and environmental factors

**Figure 1:**
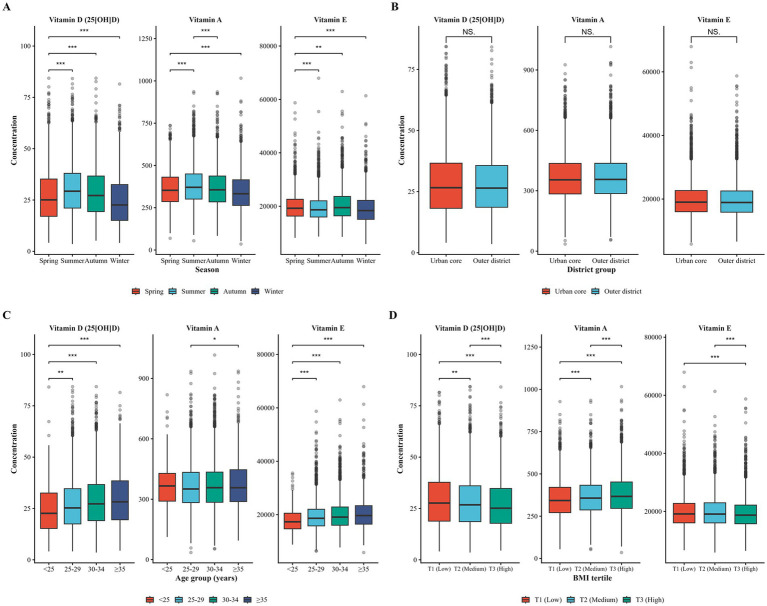
Maternal vitamin distributions by demographic and environmental factors. Boxplots display vitamin D (25[OH]D), vitamin A, and vitamin E concentrations stratified by **(A)** season, **(B)** residential district, **(C)** maternal age, and **(D)** BMI tertiles. Boxes represent the interquartile range, lines indicate medians, and whiskers extend to 1.5 × IQR. Seasonal variation: Vitamin D peaked in summer (29.3 ng/mL) compared to winter (22.6 ng/mL, *p* < 0.001); vitamin A showed similar patterns. Age: vitamin D increased with age (22.6 to 28.2 ng/mL, *p* < 0.001); vitamin E excess increased from 29.0 to 47.7% (*p* < 0.001). BMI: Inverse relationship with vitamin D; positive with vitamin A. Statistical comparisons: Group differences were assessed using pairwise Wilcoxon rank-sum tests for all possible pairwise combinations within each stratification variable. *p*-values were adjusted for multiple comparisons using the Bonferroni correction (season and age groups: 6 pairwise comparisons each; residential district: 1 comparison; BMI tertiles: 3 pairwise comparisons). Asterisks indicate Bonferroni-adjusted significance levels: *p < 0.05, **p < 0.01, ***p < 0.001. Only statistically significant pairwise comparisons after Bonferroni correction are displayed.

Comprehensive stratified distributions are presented in Supplementary Tables S1, S2 and Figure 1. All three vitamins exhibited significant seasonal variation (all *p* < 0.001; Figure 1A). Vitamin D showed the most pronounced seasonal gradient, with substantially higher concentrations in summer and the poorest status in winter. Specifically, the median 25(OH)D concentration declined from 29.3 ng/mL in summer to 22.6 ng/mL in winter, accompanied by an increase in deficiency prevalence from 21.6 to 42.4%. Vitamin A followed a similar but less marked seasonal pattern, with winter showing the highest deficiency prevalence (approximately 10%, compared with 3.8% in summer). In contrast, vitamin E excess demonstrated a distinct seasonal profile, peaking in autumn (46.6%) and remaining lower in summer and winter (approximately 38%). Residential location was not associated with vitamin status, with no significant differences observed between women residing in urban core and outer district areas (Figure 1B). In contrast, marked age-related trends were evident, particularly for vitamin D and vitamin E (Figure 1C). Vitamin D concentrations increased progressively with age, with deficiency prevalence declining from 41.7% in women aged <25 years to 26.2% in those aged ≥35 years. Conversely, vitamin E excess increased sharply across age categories, rising from 29.0 to 47.7% over the same age range. Vitamin A concentrations did not differ significantly by age. Body mass index (BMI) was differentially associated with vitamin status. Higher BMI was associated with lower vitamin D concentrations (median 25.1 ng/mL in the highest tertile vs. 27.6 ng/mL in the lowest) and a modestly higher prevalence of deficiency. In contrast, vitamin A concentrations increased with BMI, with deficiency decreasing from 6.9% in the lowest tertile to 4.1% in the highest. Vitamin E excess showed only modest variation across BMI tertiles. Overall, these stratified analyses highlight clear vitamin-specific patterns, with vitamin D showing the greatest sensitivity to environmental and demographic factors, vitamin A exhibiting relatively modest variation, and vitamin E excess predominantly structured by age and season rather than by adiposity or residence.

### Meteorological determinants and interactive effects

**Figure 2:**
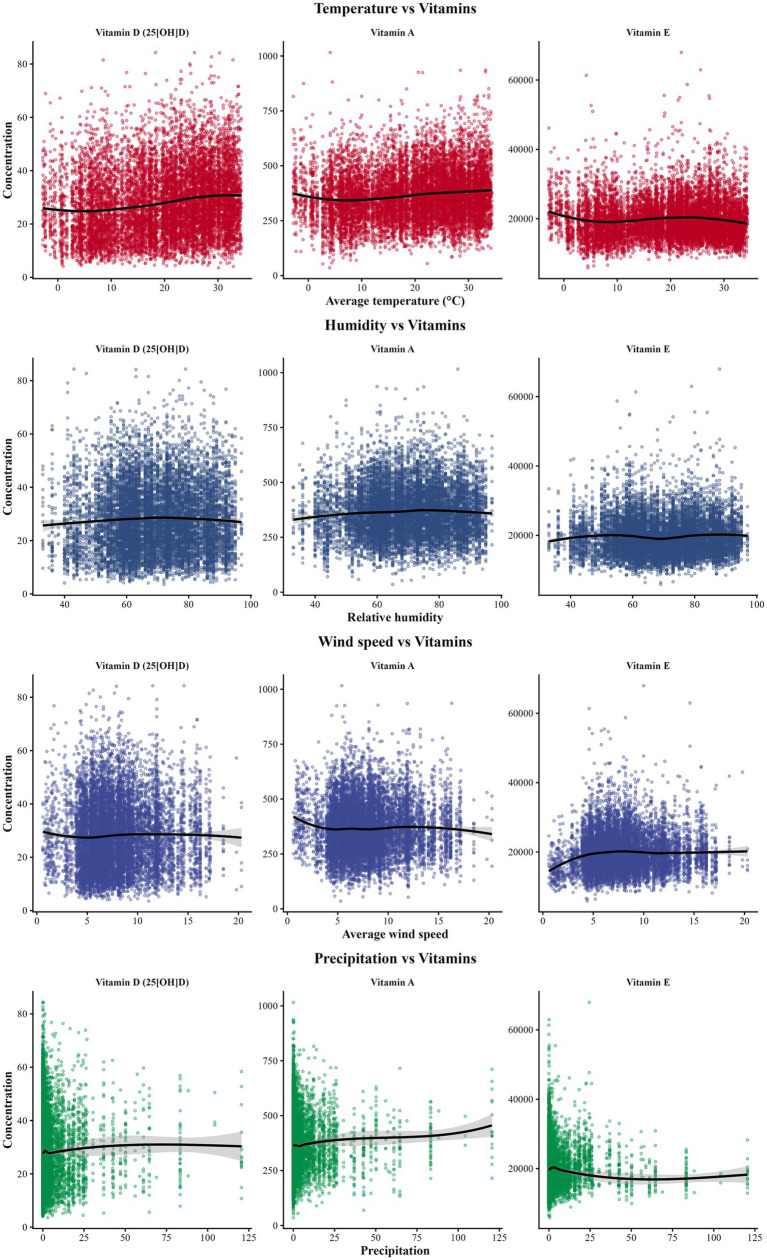
Weather factors and vitamin concentrations. Scatter plots with LOESS curves show relationships between meteorological variables and vitamins. Temperature showed the strongest statistical associations (vitamin D: *ρ* = 0.184; vitamin A: ρ = 0.125; both *p* < 0.001). Humidity, wind speed, and precipitation demonstrated weaker associations (ρ = 0.011–0.057). Points: *α* = 0.35 transparency. All measurements were taken at 37–40 weeks gestation.

Temperature emerged as the strongest meteorological correlate of vitamin status (Supplementary Figure S1). Spearman correlations were *ρ* = 0.184 for vitamin D and ρ = 0.125 for vitamin A (both *p* < 0.001), while vitamin E showed no significant correlation (ρ = 0.004, *p* = 0.652). Stratification by temperature tertiles showed vitamin D deficiency declined from 39.8% in the lowest tertile to 21.2% in the highest (Supplementary Table S1). In contrast, other meteorological variables showed only weak correlations with vitamins D and A. Scatter plots with locally weighted regression indicated subtle non-linear relationships, with temperature showing the most pronounced positive associations (Figure 2).

Univariate logistic regression analyses identified temperature as the strongest individual predictor of vitamin deficiencies (Supplementary Table S3). Each 1 °C increase in temperature was associated with a reduced risk of vitamin D deficiency and vitamin A deficiency (both *p* < 0.001). Humidity also showed significant univariate associations with vitamin D deficiency (*p* = 0.005) and vitamin A deficiency (*p* = 0.004). Wind speed was associated with vitamin D deficiency (*p* = 0.001) and vitamin E excess (*p* = 0.005) in univariate models. Precipitation showed marginal associations with vitamin D deficiency (*p* = 0.052) but not with vitamin A or E abnormalities. Multivariable logistic regression models adjusting for all meteorological variables, maternal age, BMI, season, and district confirmed temperature as the dominant predictor (Supplementary Table S3). Each 1 °C increase in temperature was associated with a 3.0% lower risk of vitamin D deficiency (aOR = 0.970, 95% CI 0.962–0.979, *p* < 0.001) and a 2.4% lower risk of vitamin A deficiency (aOR = 0.976, 95% CI 0.959–0.994, *p* = 0.008), while other meteorological effects became non-significant. Maternal age ≥25 years conferred substantial protection against vitamin D deficiency (aOR = 0.475–0.707 vs. <25 years; all *p* < 0.003). Higher BMI increased the risk of vitamin D deficiency (aOR = 1.244 for T3 vs. T1, *p* < 0.001) but decreased the risk of vitamin A deficiency (aOR = 0.557, *p* < 0.001). Vitamin E excess increased progressively with age (aOR = 2.311 for ≥35 vs. <25 years, *p* < 0.001).

**Figure 3:**
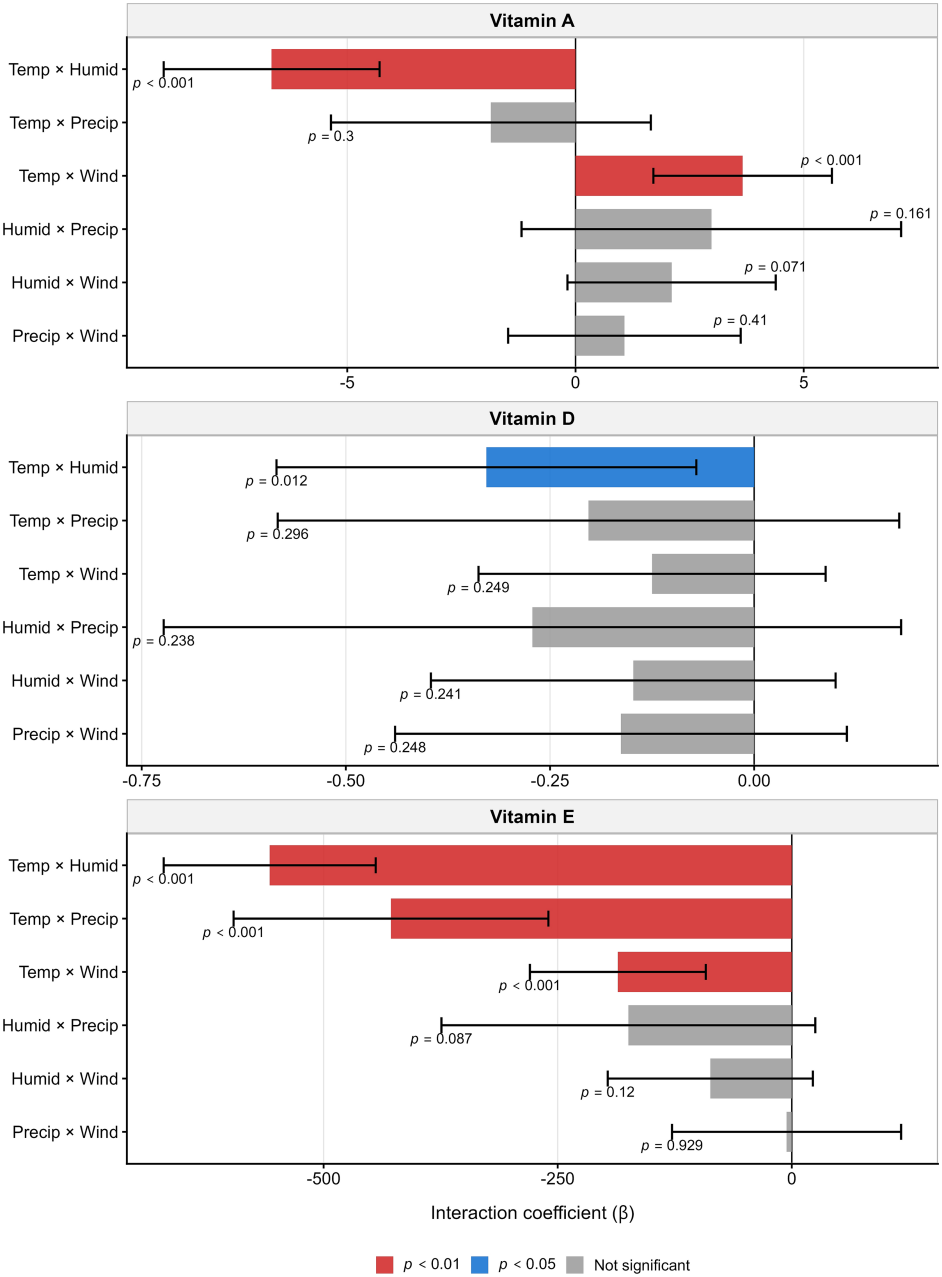
Weather factor interactions on vitamins. Standardized regression coefficients (*β*, 95% CI) for meteorological interactions. Models were adjusted for maternal age, BMI, gestational age, gravidity, parity, season, and district. Vitamin A: Temperature × humidity (β = −4.52, *p* < 0.001); temperature × wind (β = 3.87, *p* < 0.001). Vitamin D: Temperature × humidity (β = 0.18, *p* = 0.012). Vitamin E: Multiple significant interactions including temperature × humidity (β = 478.6), temperature × precipitation (β = −289.4), and temperature × wind (β = 156.8; all *p* < 0.001). Colors: red (*p* < 0.01), blue (*p* < 0.05), gray (NS).

Standardized interaction models identified statistically significant multiplicative effects between meteorological variables (Figure 3). For vitamin A, the temperature × humidity (*β* = −4.52, *p* < 0.001) and temperature × wind (β = 3.87, *p* < 0.001) interactions were highly significant. Vitamin D showed a significant temperature × humidity interaction (β = 0.18, *p* = 0.012). Vitamin E demonstrated substantial interactions: temperature × humidity (β = 478.6), temperature × precipitation (β = −289.4), and temperature × wind (β = 156.8; all *p* < 0.001), indicating significant effect modification of temperature–vitamin associations by concurrent meteorological conditions.

### Associations between maternal vitamin status and delivery/neonatal outcomes

**Figure 4:**
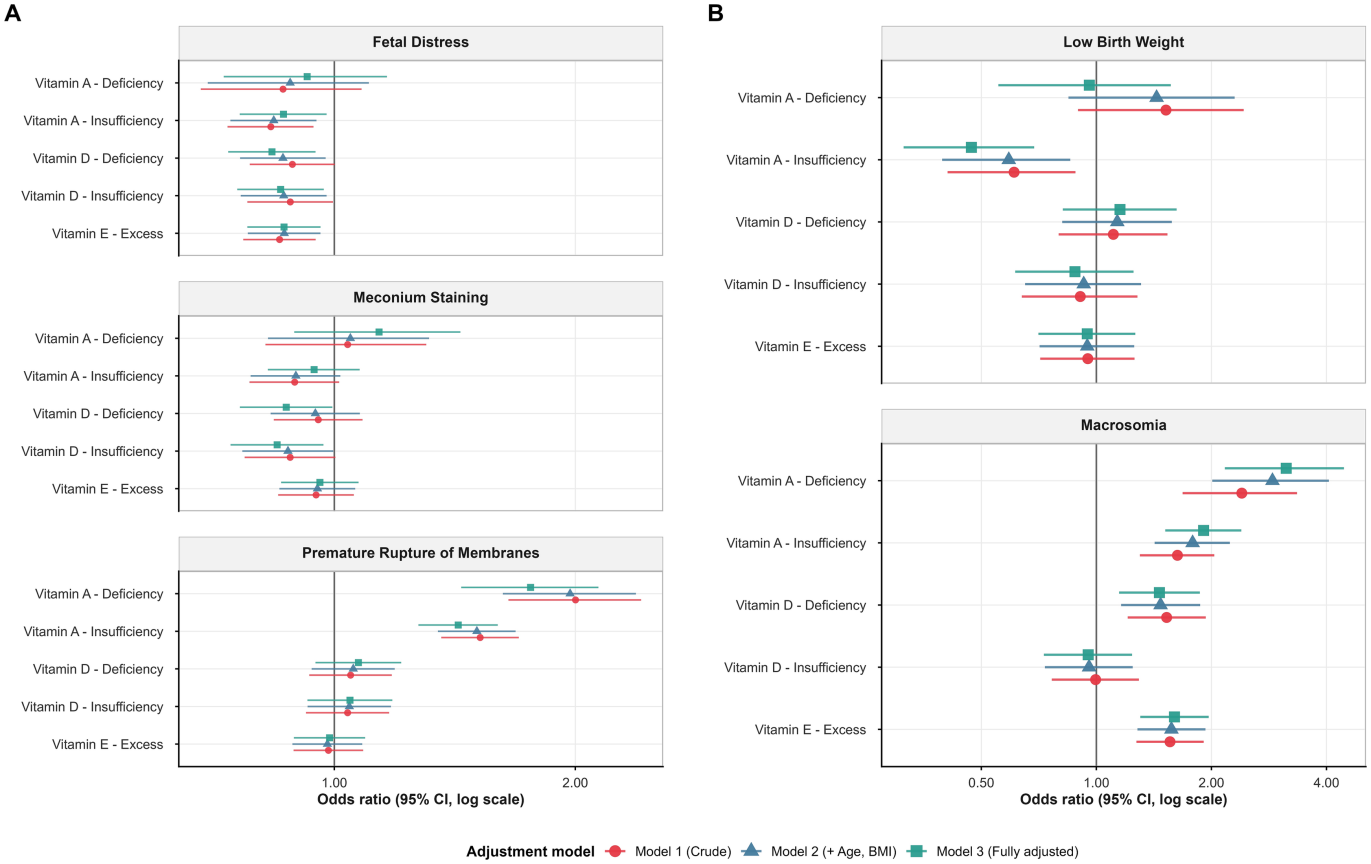
Forest plot: vitamin status and delivery/neonatal outcomes. Adjusted odds ratios (95% CI) from sequential logistic regression for five perinatal outcomes. **(A)** Presents delivery-related outcomes: fetal distress, meconium-stained amniotic fluid, and premature rupture of membranes (PROM). **(B)** Presents neonatal growth outcomes: low birth weight and macrosomia. Three sequential models are shown: Model 1 (crude, red circles); Model 2 (adjusted for maternal age and BMI, blue triangles); Model 3 (fully adjusted for age, BMI, season, residential district, and gestational age, green squares).

**Figure 5:**
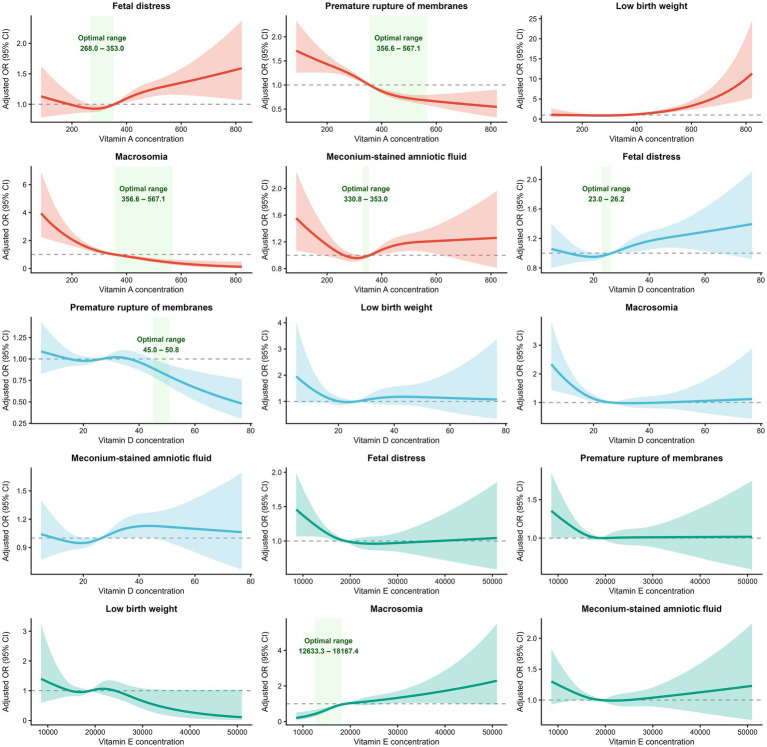
Dose–response relationships between maternal vitamin concentrations and perinatal outcomes. Dose–response associations between continuous maternal concentrations of vitamins A, D, and E and five perinatal outcomes were examined using restricted cubic spline (RCS) regression models. Curves represent adjusted odds ratios (aORs), and shaded bands indicate 95% confidence intervals. All models were adjusted for maternal age, body mass index, season, residential district, and gestational age at blood sampling. The horizontal dashed line denotes aOR = 1.0. For vitamin A, a monotonic exposure–response pattern was observed for macrosomia (*p*_overall_ < 0.001, *p*_nonlinear_ = 0.666) and for premature rupture of membranes (*p*_overall_ < 0.001, *p*_nonlinear_ = 0.182), whereas a non-linear (U-shaped) association was observed for low birth weight (*p*_nonlinear_ = 0.015). For vitamin D, a non-linear association with macrosomia was observed (*p*_nonlinear_ = 0.023), while associations with fetal distress and meconium-stained amniotic fluid showed lower estimated odds across intermediate concentration ranges without evidence of non-linearity. For vitamin E, a threshold-like association with macrosomia was observed (*p*_nonlinear_ = 0.020), with higher estimated odds at supraphysiological concentrations. Green shaded areas (where shown) indicate statistically supported “optimal ranges,” defined as continuous concentration intervals associated with lower estimated odds (aOR < 1.0) with 95% confidence intervals not crossing unity. Optimal ranges were annotated only for vitamin–outcome pairs meeting this statistical criterion. No optimal range was indicated for outcomes in which this condition was not satisfied. Detailed numerical estimates of optimal ranges are provided in Supplementary Table S5.

Sequential multivariable logistic regression demonstrated consistent associations between maternal vitamin status and major delivery and neonatal outcomes (Table 2; Figures 4, 5). For fetal distress (n = 1,785; 16.5%), both vitamin A insufficiency and vitamin D insufficiency were associated with lower odds (aOR = 0.86, 95% CI 0.76–0.98, *p* = 0.022; aOR = 0.86, 95% CI 0.76–0.97, *p* = 0.015), and vitamin E excess was likewise associated with lower odds of fetal distress (aOR = 0.87, 95% CI 0.78–0.96, *p* = 0.007). Restricted cubic spline (RCS) analyses showed largely monotonic exposure–response relationships, with no statistically significant evidence of nonlinearity (*p*_nonlinear_ > 0.05). Within the observed exposure range, selected concentration intervals associated with relatively lower estimated odds were identifiable for vitamin A and vitamin D (Figure 5; Supplementary Table S4).

**Table 2:** Associations between maternal vitamin status and delivery and neonatal outcomes.

Type	Outcome	Vitamin	Status	aOR (95% CI)	*p* value	Events
Delivery	Fetal distress	Vitamin A	Insufficiency	0.86 (0.76–0.98)	0.022	1785
	Fetal distress	Vitamin A	Deficiency	0.92 (0.73–1.16)	0.513	1785
	Fetal distress	Vitamin D	Insufficiency	0.86 (0.76–0.97)	0.015	1785
	Fetal Distress	Vitamin D	Deficiency	0.84 (0.74–0.95)	0.005	1785
	Fetal distress	Vitamin E	Excess	0.87 (0.78–0.96)	0.007	1785
	Meconium staining	Vitamin A	Insufficiency	0.94 (0.83–1.08)	0.388	1,576
	Meconium staining	Vitamin A	Deficiency	1.14 (0.89–1.44)	0.291	1,576
	Meconium staining	Vitamin D	Insufficiency	0.85 (0.74–0.97)	0.016	1,576
	Meconium staining	Vitamin D	Deficiency	0.87 (0.76–0.99)	0.041	1,576
	Meconium Staining	Vitamin E	Excess	0.96 (0.86–1.07)	0.465	1,576
	Premature rupture of membranes	Vitamin A	Insufficiency	1.43 (1.27–1.60)	<0.001	1926
	Premature rupture of membranes	Vitamin A	Deficiency	1.76 (1.44–2.14)	<0.001	1926
	Premature rupture of membranes	Vitamin D	Insufficiency	1.05 (0.93–1.18)	0.47	1926
	Premature rupture of membranes	Vitamin D	Deficiency	1.07 (0.95–1.21)	0.271	1926
	Premature rupture of membranes	Vitamin E	Excess	0.99 (0.89–1.09)	0.793	1926
Neonatal	Low birth weight	Vitamin A	Insufficiency	0.47 (0.31–0.69)	<0.001	202
	Low birth weight	Vitamin A	Deficiency	0.96 (0.55–1.57)	0.872	202
	Low birth weight	Vitamin D	Insufficiency	0.88 (0.61–1.25)	0.477	202
	Low birth weight	Vitamin D	Deficiency	1.15 (0.82–1.62)	0.415	202
	Low birth weight	Vitamin E	Excess	0.95 (0.71–1.26)	0.712	202
	Macrosomia	Vitamin A	Insufficiency	1.91 (1.51–2.39)	<0.001	391
	Macrosomia	Vitamin A	Deficiency	3.14 (2.17–4.44)	<0.001	391
	Macrosomia	Vitamin D	Insufficiency	0.95 (0.73–1.24)	0.717	391
	Macrosomia	Vitamin D	Deficiency	1.46 (1.15–1.87)	0.002	391
	Macrosomia	Vitamin E	Excess	1.60 (1.30–1.97)	<0.001	391

Adjusted odds ratios (aORs) with 95% confidence intervals were derived from multivariable logistic regression models adjusting for maternal age, body mass index, season, district, and gestational age at blood collection. Shaded rows indicate neonatal outcomes.

For meconium-stained amniotic fluid (*n* = 1,576; 14.6%), categorical analyses indicated lower odds among women with vitamin D deficiency or insufficiency (aOR = 0.87 and 0.85, respectively; both *p* < 0.05). In RCS models, vitamin A demonstrated a statistically significant nonlinear association (*p*_overall_ = 0.014; *p*_nonlinear_ = 0.012; Figure 5 and Supplementary Table S4), characterized by a mild U-shaped pattern. A concentration range associated with lower estimated odds could be defined for vitamin A, whereas no statistically supported optimal range was identified for vitamin D or vitamin E (Figure 5; Supplementary Table S4). For premature rupture of membranes (PROM) (n = 1,926; 17.8%), vitamin A insufficiency (aOR = 1.43, 95% CI 1.27–1.60) and deficiency (aOR = 1.76, 95% CI 1.44–2.14) were associated with higher odds in categorical models. RCS analyses confirmed a strong overall association for vitamin A (*p*_overall_ < 0.001) without evidence of nonlinearity (*p*_nonlinear_ = 0.182), indicating a monotonic exposure–response pattern. Concentration ranges associated with lower estimated odds were identifiable for vitamin A and, to a lesser extent, for vitamin D within its physiological distribution (Figure 5; Supplementary Table S4). For low birth weight (LBW) (*n* = 202; 1.9%), vitamin A insufficiency was associated with lower odds in categorical analyses (aOR = 0.47, 95% CI 0.31–0.69; *p* < 0.001), while vitamin A deficiency and sufficiency were not statistically significant. RCS analyses demonstrated a statistically significant non-linear exposure–response relationship (*p*_overall_ < 0.001; *p*_nonlinear_ = 0.015), with the lowest estimated odds occurring at intermediate concentrations. However, the 95% confidence intervals overlapped unity across the exposure range, precluding identification of a statistically supported optimal concentration interval (Figure 5; Supplementary Table S4). For macrosomia (*n* = 391; 3.6%), higher odds were observed in association with vitamin A insufficiency (aOR = 1.91, 95% CI 1.51–2.39), and vitamin A deficiency, which was strongly associated with macrosomia (aOR = 3.14, 95% CI 2.17–4.44), as well as with vitamin E excess (aOR = 1.60, 95% CI 1.30–1.97). In RCS analyses, vitamin A showed an exposure–response pattern in which higher physiological concentrations were associated with lower estimated odds, whereas vitamin E exhibited a threshold-like association, with elevated odds observed at higher concentrations. For vitamin D, a U-shaped pattern was observed, but no concentration interval met confidence-interval criteria for defining a statistically supported optimal range (Figure 5; Supplementary Table S4).

Overall, categorical and spline-based analyses yielded directionally consistent associational patterns across outcomes (Table 2; Figures 4, 5). Delivery-related outcomes (fetal distress, meconium-stained amniotic fluid, and PROM) tended to show lower estimated odds within specific ranges of maternal vitamin A and D concentrations, whereas neonatal growth outcomes—particularly macrosomia—were more strongly associated with higher concentrations of vitamins A and E. The annotated spline plots highlight concentration intervals associated with lower estimated odds where supported by confidence-interval criteria, while avoiding over-interpretation for vitamin–outcome pairs lacking statistically defensible ranges.

## Discussion

Abnormalities in maternal fat-soluble vitamin status during late pregnancy were common in this cohort and were consistently associated with delivery and neonatal outcomes in central China. First, we simultaneously examined three fat-soluble vitamins with comprehensive meteorological integration, revealing temperature as the strongest environmental correlate (3.0% lower vitamin D deficiency risk per 1 °C increase in multivariable models) with significant interaction terms (temperature × humidity and temperature × wind), indicating that the associations between temperature and vitamin status differed according to concurrent humidity and wind conditions. Second, we identified a striking threefold higher odds of macrosomia associated with vitamin A deficiency—a relationship inadequately characterized in previous research—alongside an inverse association between vitamin A insufficiency and low birth weight observed in categorical analyses, which should be interpreted within a broader pattern of non-linear exposure–response relationships rather than as evidence of biological protection. Third, we demonstrated that 41.8% of women exhibited vitamin E excess, which was associated with elevated macrosomia risk, challenging the conventional focus on deficiency prevention alone and revealing a bidirectional risk profile that requires optimization rather than maximization of vitamin status. These findings provide the first systematic characterization of meteorological determinants across multiple vitamins and highlight the urgent need for population-specific evidence to inform nutritional strategies in subtropical Asian populations. Consistent with prior observational literature, vitamin D deficiency was associated with higher odds of macrosomia, highlighting the relevance of maternal vitamin D status to fetal growth outcomes (33).

Our observed vitamin D deficiency prevalence (30.2%) is substantially lower than previously reported rates in northern Chinese populations (2, 3) and closely matches rates documented in other subtropical and tropical regions globally (4, 21), reflecting greater year-round UVB exposure at lower latitudes. This geographic variability in maternal vitamin D status (4) highlights the need for latitude-specific reference ranges and supplementation protocols rather than uniform global guidelines. The 10,824-participant cohort from Wuhan (30°N) provides critical data that fill a gap in the literature for inland subtropical Asian populations.

In addition to geographic variation, vitamin D status was associated with demographic factors. The inverse association between BMI and vitamin D status observed in our cohort (32.6% deficiency in the highest vs. 28.0% in the lowest BMI tertile) aligns with well-established pathophysiological mechanisms (13, 34). Obesity-associated vitamin D deficiency may involve multiple pathways: (1) sequestration of lipophilic 25(OH)D in expanded adipose tissue compartments, reducing bioavailable circulating concentrations; (2) volumetric dilution in larger body pools; and (3) potentially decreased cutaneous synthesis due to reduced outdoor physical activity (13, 34). This relationship underscores the need for BMI-adjusted supplementation protocols, as obese pregnant women require higher doses to achieve equivalent serum 25(OH)D concentrations compared to normal-weight women.

Beyond BMI, age-related and seasonal patterns in vitamin status reflect distinct behavioral mechanisms aside from meteorological factors alone. The higher vitamin D deficiency in younger women aligns with contemporary lifestyle patterns documented across Asian populations, where studies have reported deficiency prevalence exceeding 90% among young women attributed to indoor occupations, sun avoidance behaviors for skin protection, and reduced outdoor activities (35–37). Winter vitamin D nadir (22.6 ng/mL) likely reflects both reduced UVB availability and behavioral thermoregulation through increased clothing coverage and indoor preference during cold periods. The absence of urban–rural differences in our cohort contrasts with studies documenting higher urban deficiency rates (38), suggesting relatively homogeneous lifestyle patterns and sun exposure behaviors throughout our study region, possibly reflecting advanced urbanization where outdoor agricultural work is uncommon and healthcare access is equitable across residential areas.

The strong association between vitamin A deficiency and macrosomia with steep dose–response gradients represents our most clinically significant and novel finding. While excessive vitamin A intake during early pregnancy is associated with congenital malformations (8), the relationship between deficiency and macrosomia remains poorly characterized. Vitamin A has been shown to play critical roles in placental development and nutrient transport in experimental studies (39); deficiency may be associated with altered placental function, though whether this represents a causal mechanism or reflects confounding requires further investigation. Retinoic acid, the active metabolite of vitamin A, has been shown in experimental studies to regulate expression of genes involved in glucose homeostasis and insulin signaling in the placenta, though the relevance of this pathway to the observed associations in our population-based study remains unclear (40). These mechanistic interpretations are speculative, as placental function, glucose metabolism, and dietary intake were not directly measured in this study.

The observed inverse association between vitamin A insufficiency and low birth weight in categorical analyses was compatible with non-linear exposure–response patterns; however, restricted cubic spline analyses showed confidence intervals crossing unity across the exposure range, precluding inference of a protective or biologically optimal concentration. For vitamin D and macrosomia, spline curves were compatible with a U-shaped pattern with a nadir near the mid-range of observed concentrations, but no confidence-interval–supported optimal range could be defined, consistent with prior observational studies reporting non-linear associations between maternal 25(OH)D and fetal growth outcomes (41). For vitamin E, a threshold-like association was observed, with higher concentrations associated with increased odds of macrosomia. This finding is notable given that 41.8% of women in the cohort exceeded 20,000 ng/mL. However, because detailed data on dietary intake and supplement use were unavailable, these associations should be interpreted cautiously and do not permit inference regarding optimal intake levels during pregnancy.

These patterns suggest that maternal vitamin optimization may require balancing risks across multiple perinatal outcomes rather than simply preventing deficiency. However, several critical limitations preclude definitive determination of optimal ranges from our data. First, cross-sectional assessment at a single gestational timepoint cannot establish whether the observed concentrations represent stable maternal status or reflect dynamic changes related to fetal demands, as vitamin status demonstrates substantial longitudinal variation from preconception through pregnancy that cannot be inferred from single-timepoint measurements (42). Second, optimal ranges may differ across perinatal outcomes (e.g., the vitamin A concentration minimizing low birth weight risk may differ from that minimizing macrosomia risk). Third, population heterogeneity in genetics, dietary patterns, and supplement use likely results in individual-specific rather than universal optimal ranges (43). Prospective cohort studies with serial measurements across all trimesters, coupled with randomized dose-finding trials stratified by baseline vitamin status and individual characteristics, are essential to validate these preliminary observations and establish evidence-based supplementation protocols for subtropical Asian populations. This pattern underscores the complexity of maternal nutritional optimization beyond simple deficiency prevention.

The apparent “protective” association between vitamin A insufficiency and low birth weight warrants cautious interpretation and should not inform clinical practice. This counter-intuitive finding likely reflects methodological artifacts rather than genuine biological benefit. Five alternative explanations merit consideration: First, residual confounding from unmeasured socioeconomic factors, dietary patterns, and supplement use may simultaneously influence vitamin A status and birth weight (44). Second, survival bias could selectively exclude pregnancies with both severe insufficiency and growth restriction, as such fetuses may experience earlier pregnancy loss or preterm delivery (excluded from our ≥37-week cohort). Third, the U-shaped dose–response demonstrated by restricted cubic spline analysis, together with overlapping confidence intervals across the exposure range, indicates that optimal vitamin A status lies within a physiological range rather than insufficiency being beneficial; categorical analyses may therefore reflect statistical artifacts at curve extremes. Fourth, reverse causation cannot be excluded, as maternal vitamin status at 37–40 weeks may be influenced by pre-existing fetal growth patterns through placental adaptations. Finally, chance findings remain possible despite statistical significance, as multiple comparisons across three vitamins, five outcomes, and three exposure categories increase the probability of spurious associations (45). These considerations indicate that vitamin A insufficiency should not be interpreted as protective. Clinical recommendations should continue emphasizing adequate vitamin A intake through balanced diet and appropriate supplementation rather than targeting insufficiency. Future prospective studies with serial measurements, comprehensive dietary assessment, and standardized outcome ascertainment are needed to definitively characterize optimal vitamin A ranges during pregnancy.

In addition to neonatal growth outcomes, delivery-related events also showed consistent associations with maternal vitamin status. Vitamin A deficiency was associated with higher odds of premature rupture of membranes. While experimental studies suggest vitamin A influences epithelial integrity, collagen metabolism, and oxidative balance (46, 47), whether these mechanisms underlie the observed association or reflect residual confounding cannot be determined from our cross-sectional data. Conversely, low to moderate levels of vitamins D and A were associated with lower risks of fetal distress and meconium-stained amniotic fluid, though the mechanisms underlying these associations remain unclear and require investigation in prospective studies (48). Although these associations were modest in magnitude, they suggest that maternal vitamin homeostasis may influence both the structural and functional stability of the delivery process, complementing its well-established role in regulating fetal growth (49).

Our finding that 41.8% exhibited vitamin E excess with associated macrosomia risk aligns with recent Chinese research demonstrating increased odds of large-for-gestational-age with elevated *α*-tocopherol (7). Mechanisms underlying vitamin E excess-associated macrosomia remain incompletely understood but may involve multiple pathways. At supraphysiological concentrations, α-tocopherol has been shown in experimental studies to exhibit pro-oxidant rather than antioxidant properties (34, 50, 51). However, whether such mechanisms explain the observed association with macrosomia cannot be inferred from the present epidemiological data and may also reflect confounding by unmeasured dietary and supplementation patterns. Experimental studies have demonstrated that oxidative stress can affect placental function and fetal growth through effects on trophoblast invasion, vascular remodeling, and nutrient transfer, though the relevance of this pathway to the observed statistical associations in our population-based study remains speculative (51). Essential micronutrients, including fat-soluble vitamins, are known from experimental studies to function optimally within narrow physiological ranges (40). Whether deviations from these ranges causally contribute to adverse outcomes or simply correlate with other causal factors remains uncertain in observational studies. Additionally, excessive vitamin E has been shown in some studies to potentially affect the absorption and metabolism of other fat-soluble vitamins, particularly vitamin K. However, whether this contributes to the observed associations requires investigation.

The high prevalence of vitamin E excess observed in this cohort may reflect a combination of routine prenatal supplementation, dietary patterns, their interaction, or other unmeasured factors, rather than supplementation alone. In urban China, prenatal multivitamin use is common, and when combined with dietary sources such as vegetable oils, nuts, and fortified foods, total vitamin E intake may exceed physiological requirements. However, because individual-level data on supplement use and detailed dietary intake were unavailable, the relative contribution of these factors cannot be disentangled. Accordingly, the observed association between vitamin E excess and macrosomia should be interpreted with caution, and causal inference cannot be established. Taken together, these findings suggest that both deficient and excessive vitamin E status may be associated with unfavorable pregnancy outcomes, underscoring the importance of avoiding indiscriminate supplementation and the need for future studies incorporating detailed dietary assessment and supplement-use data to inform population-specific nutritional recommendations.

Beyond individual vitamin-outcome associations, understanding the environmental determinants of maternal vitamin status is critical for designing effective interventions. Our systematic meteorological analysis identifies temperature as the primary environmental correlate, with each 1 °C increase associated with a 3.0% lower risk of vitamin D deficiency and a 2.4% lower risk of vitamin A deficiency. Previous studies in subtropical China have shown similar temperature-vitamin D correlations (11), but our findings extend these observations by: (1) demonstrating temperature effects on vitamin A; (2) identifying significant meteorological interactions―temperature × humidity and temperature × wind speed―indicating significant effect modification, such that the associations between temperature and vitamin status differed under concurrent atmospheric conditions; and (3) revealing that vitamin E excess peaks in autumn (46.6%) compared to summer and winter (38.4–38.8%), suggesting season-specific dietary or metabolic patterns.

Temperature emerged as the predominant environmental correlate, and the observed non-linear relationships and interaction terms are compatible with combined biological and behavioral influences. The strong temperature-vitamin D association observed in our study is consistent with multiple plausible mechanisms, including enhanced cutaneous vitamin D3 synthesis through increased UV-B exposure (13, 14), behavioral changes such as reduced clothing and increased outdoor activity, and altered dietary patterns during warmer periods (12, 52). Dietary patterns in meat-eaters versus vegetarians significantly influence vitamin D status (14), and similar dietary shifts may occur seasonally. Importantly, because meteorological variables were standardized, interaction coefficients reflect relative effect modification rather than absolute changes in vitamin concentrations. These interaction effects suggest heterogeneity in associations across climatic contexts, rather than uniform temperature effects. The observed temperature-humidity interaction may operate through both physical and behavioral mechanisms, though the relative contributions of each cannot be disentangled in this observational study. Behaviorally, thermal discomfort from combined heat and humidity may discourage outdoor activity, with studies documenting paradoxical seasonal patterns where vitamin D levels are lower in summer than winter in hot, humid climates (37, 53).

The association between temperature and vitamin D status exhibited threshold-dependent patterns in previous large cohort studies, 25(OH)D concentration changes were minimal below 20 °C, showed maximum variation between 20–25 °C when outdoor activity was most comfortable, and then declined above 25 °C as thermal discomfort increased (11). Combined climatic conditions consistently outperform single-factor models in predicting maternal vitamin D status across subtropical populations, but the relative importance of specific factors varies by climate zone. In a population-based study of 1,502 pregnant women in Taiwan, the determinant hierarchy reversed between climate zones: southern tropical regions showed sunlight factors exceeding dietary factors, while northern subtropical regions exhibited dietary factors exceeding sunlight factors (12). Multiple studies across diverse geographic settings have confirmed that vitamin D deficiency relates to combined environmental factors rather than any single predictor (54).

These findings suggest that integrated climatic condition assessment may help identify periods of elevated deficiency risk and inform geographically-tailored intervention strategies. Importantly, seasonal variation in vitamins A and E may also be strongly influenced by season-dependent changes in food availability and dietary composition (e.g., intake of liver/egg/dairy and carotenoid-rich fruits and vegetables for vitamin A, and vegetable oils, nuts, and seeds for vitamin E), rather than meteorological factors alone (55, 56). Because detailed dietary and supplement data were unavailable, we cannot disentangle direct meteorological effects from seasonal dietary changes, and the observed seasonality should be interpreted as reflecting a combination of environmental and behavioral factors. From a public health perspective, these temperature-by-humidity, temperature-by-season, and climate zone-by-predictor interactions demonstrate that vitamin supplementation strategies should consider local meteorological patterns and population-specific seasonal variations rather than relying solely on calendar-based seasonal categorizations (20). Enhanced nutritional surveillance is particularly warranted during winter months and in regions characterized by low ambient temperatures combined with high humidity, where vitamin deficiency risk is substantially elevated.

Study strengths include large sample size, standardized HPLC vitamin measurements with rigorous quality control, daily meteorological monitoring on individual blood collection dates, comprehensive outcome ascertainment, and sequential regression models. However, several limitations should be considered. First, the cross-sectional assessment of vitamin concentrations in late pregnancy precludes causal inference and raises the possibility of reverse causation, particularly for fetal growth outcomes such as macrosomia. Second, single measurements at 37–40 weeks do not capture temporal variability across pregnancy. In addition, meteorological exposures were assessed using daily values on the date of blood collection, which may not fully capture the biologically relevant exposure window for vitamin D status that reflects cumulative environmental conditions over preceding weeks, potentially leading to exposure misclassification and attenuation of observed associations. Third, and representing a primary limitation, information on dietary intake and supplement use was unavailable, which may result in substantial residual confounding. Dietary patterns and supplementation practices likely vary systematically with socioeconomic and health-seeking behaviors that independently influence perinatal outcomes, and the absence of these data particularly precludes definitive attribution of the observed vitamin E excess or its associations with pregnancy outcomes. Additionally, data on gestational diabetes and hypertensive disorders were not available, further limiting our ability to adjust for important pregnancy complications. Fourth, although vitamin status categories were defined using widely accepted guideline-based cut-offs, these thresholds have not been specifically validated for late-pregnancy women in subtropical Chinese populations, and population-specific optimal ranges may differ. Finally, the hospital-based, single-center urban sample may limit generalizability to rural or other regional populations with different environmental exposures, dietary patterns, and healthcare access. Future studies using prospective cohort designs with serial vitamin measurements across pregnancy, together with biologically meaningful averaging or lag-based assessment of meteorological exposures (e.g., over several weeks prior to blood collection), are needed to better characterize cumulative environmental influences and identify critical intervention windows. Such approaches, potentially complemented by targeted interventional and mechanistic studies, would help clarify causal pathways and inform population-specific nutritional recommendations.

These observations are hypothesis-generating and should not be interpreted as clinical recommendations; nevertheless, several potential clinical implications warrant further investigation: (1) The 30.2% deficiency prevalence and its association with macrosomia suggests that the potential value of vitamin D screening during early pregnancy deserves evaluation in randomized trials (57, 58). Recent randomized controlled trials in diverse populations have demonstrated variable efficacy of vitamin D supplementation during pregnancy, with genetic polymorphisms in vitamin D receptor genes potentially modulating individual response to supplementation, underscoring the need for personalized approaches. (2) The strong association between vitamin A deficiency and macrosomia, as well as potential optimal effects at intermediate concentrations, suggest that vitamin A status assessment may warrant consideration, though optimal intervention strategies require investigation in randomized trials. (3) Monitoring strategies for vitamin E excess, including dietary counseling and reassessment of routine multivitamin supplementation protocols, warrant further investigation in prospective studies, particularly for formulations with high vitamin E content. (4) Our findings suggest that the integration of meteorological data into nutritional risk stratification frameworks warrants further evaluation as a hypothesis-generating approach, particularly in winter months and in regions characterized by low temperatures and high humidity.

In conclusion, abnormalities in maternal vitamins D, A, and E during late pregnancy were associated with a range of adverse delivery and neonatal outcomes. Ambient temperature emerged as a key meteorological factor associated with maternal vitamin status, with significant interactions with other environmental variables. Vitamin A deficiency showed the strongest observed association with macrosomia (approximately threefold higher odds), while excess vitamin E was associated with a 60% higher odds of macrosomia. The complex, non-linear dose–response relationships observed in this study suggest that achieving appropriate maternal vitamin status requires more than preventing deficiency alone and should consider population-specific distributions. These findings underscore the need for population-specific nutritional surveillance and prospective trials to inform evidence-based supplementation strategies in subtropical Asian populations.

## Data Availability

The original contributions presented in the study are included in the article/Supplementary material, further inquiries can be directed to the corresponding authors.

## Supplementary material

SUPPLEMENTARY FIGURE S1 Spearman correlation coefficients between meteorological factors and maternal vitamin concentrations. Heatmap displays correlation strength (ρ) between four meteorological variables (temperature, humidity, precipitation, wind speed) and serum concentrations of vitamins D, A, and E measured at 37–40 weeks gestation (*n* = 10,824). Color intensity represents correlation magnitude (dark red: strong positive; light pink: weak; white: no correlation). Temperature showed the strongest correlations with vitamin D (ρ=0.184, ****p*<0.001) and vitamin A (ρ = 0.125, ****p*<0.001), but not vitamin E (ρ = 0.004, *p* = 0.652). Humidity demonstrated weak but significant correlations with vitamins A and E (ρ = 0.032, ****p*<0.001; ρ = 0.026, ***p*<0.01). Precipitation and wind speed showed minimal associations with all vitamins (ρ = 0.011–0.057). Significance levels: **p*<0.05, ***p*<0.01, ****p*<0.001. This analysis informed the selection of meteorological variables for multivariable regression and interaction models.
