# Supplementary figures and images for: Beyond deficiency prevention: meteorological determinants and nonlinear associations of maternal vitamins D, A, and E with perinatal outcomes in 10,824 Chinese pregnancies
Source: Front Nutr. 2026 Feb 25;13:1737197. doi: 10.3389/fnut.2026.1737197 (PMC12975464; doi:10.3389/fnut.2026.1737197)

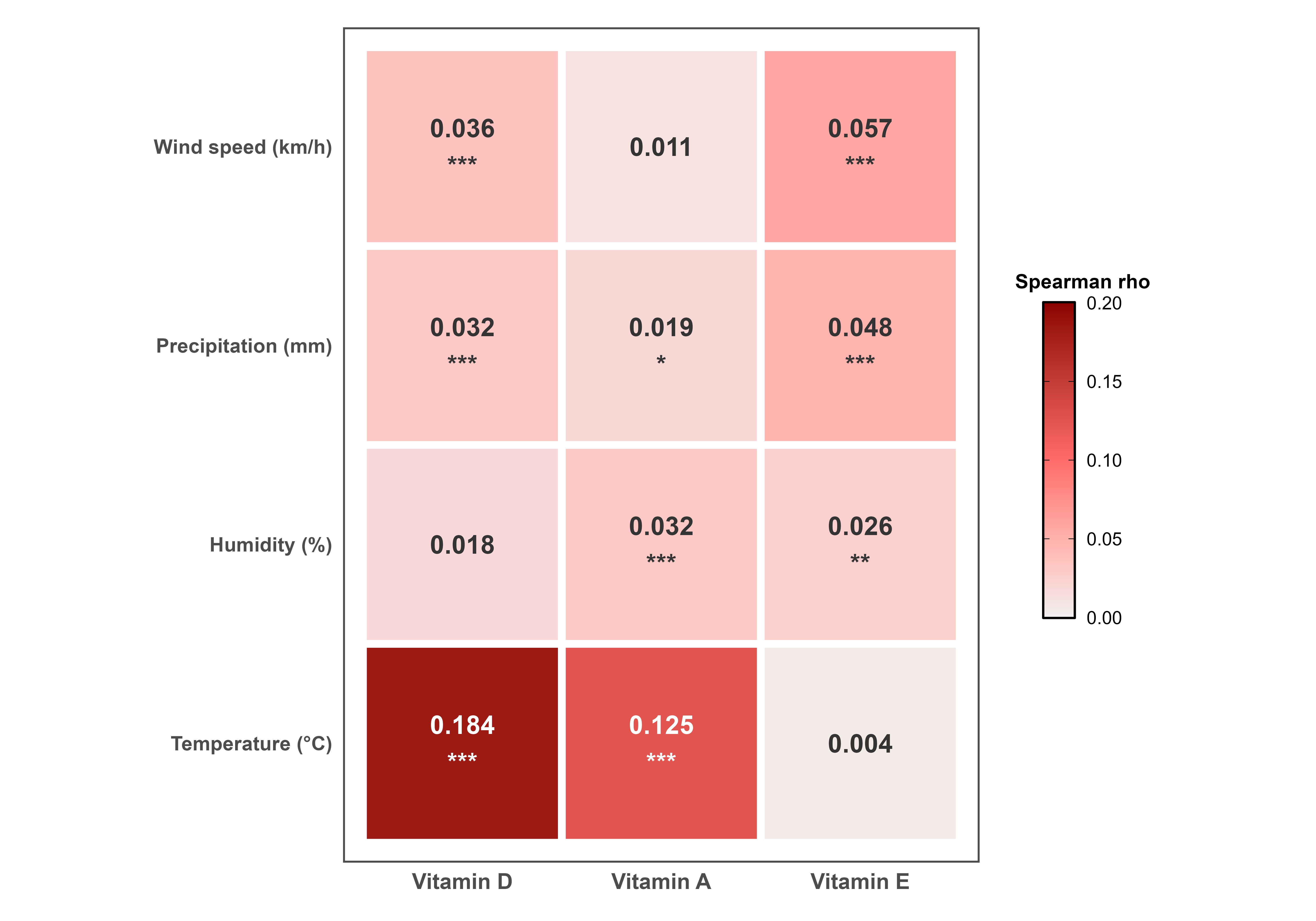

Supplement: SUPPLEMENTARY FIGURE S1 — Spearman correlation coefficients between meteorological factors and maternal vitamin concentrations. Heatmap displays correlation strength (ρ) between four meteorological variables (temperature, humidity, precipitation, wind speed) and serum concentrations of vitamins D, A, and E measured at 37–40 weeks gestation (n = 10,824). Color intensity represents correlation magnitude (dark red: strong positive; light pink: weak; white: no correlation). Temperature showed the strongest correlations with vitamin D (ρ=0.184, ***p<0.001) and vitamin A (ρ = 0.125, ***p<0.001), but not vitamin E (ρ = 0.004, p = 0.652). Humidity demonstrated weak but significant correlations with vitamins A and E (ρ = 0.032, ***p<0.001; ρ = 0.026, **p<0.01). Precipitation and wind speed showed minimal associations with all vitamins (ρ = 0.011–0.057). Significance levels: *p<0.05, **p<0.01, ***p<0.001. This analysis informed the selection of meteorological variables for multivariable regression and interaction models. [file Image_1.jpeg]
