# Supplementary material for: Beyond deficiency prevention: meteorological determinants and nonlinear associations of maternal vitamins D, A, and E with perinatal outcomes in 10,824 Chinese pregnancies
Source: Front Nutr. 2026 Feb 25;13:1737197. doi: 10.3389/fnut.2026.1737197 (PMC12975464; doi:10.3389/fnut.2026.1737197)
Supplement: Supplementary file 2 [file Table_1.docx]

| **Supplementary Table S1. Stratified Distributions of Maternal Vitamin Levels by Demographic, Residential, and Meteorological Factors** | | | | | | | | | | | |
| --- | --- | --- | --- | --- | --- | --- | --- | --- | --- | --- | --- |
| **Stratification** | **Category** | **N** | **VD Mean** | **VD Median [IQR]** | **VD Deficiency** | **VA Mean** | **VA Median [IQR]** | **VA Deficiency** | **VE Mean** | **VE Median [IQR]** | **VE Excess** |
|  |  |  | **(SD)** |  | **(%)** | **(SD)** |  | **(%)** | **(SD)** |  | **(%)** |
| Season | Spring | 2,565 | 27.1 (12.9) | 25.1 [17.1–35.2] | 34.00% | 362.5 (106.9) | 353.1 [286.6–430.9] | 4.60% | 19845 (5085) | 19246 [16324–22599] | 43.50% |
| Season | Summer | 3,305 | 30.5 (12.4) | 29.3 [21.1–38.0] | 21.60% | 381.4 (116.0) | 370.8 [301.4–449.8] | 3.80% | 19453 (5086) | 18676 [15982–22024] | 38.80% |
| Season | Autumn | 2,734 | 28.6 (12.2) | 27.2 [19.4–36.7] | 26.90% | 368.5 (115.1) | 356.0 [285.0–437.5] | 5.00% | 20447 (5900) | 19500 [16421–23665] | 46.60% |
| Season | Winter | 2,220 | 24.8 (12.2) | 22.6 [15.1–32.5] | 42.40% | 342.8 (117.0) | 332.7 [264.0–414.9] | 10.00% | 19122 (5707) | 18378 [15113–22184] | 38.40% |
| Age Group | <25 | 338 | 25.1 (12.4) | 22.6 [15.3–32.5] | 41.70% | 367.6 (111.0) | 366.0 [290.5–428.2] | 6.20% | 18104 (4840) | 17345 [14723–20553] | 29.00% |
| Age Group | 25-29 | 3,916 | 26.9 (12.3) | 25.3 [17.5–34.6] | 33.50% | 362.5 (112.8) | 350.8 [283.2–433.4] | 5.60% | 19346 (5200) | 18651 [15811–22039] | 38.80% |
| Age Group | 30-34 | 4,960 | 28.6 (12.6) | 27.2 [19.1–36.7] | 28.00% | 366.2 (115.6) | 357.4 [284.8–434.7] | 5.40% | 19875 (5450) | 19095 [16066–22886] | 43.20% |
| Age Group | ≥35 | 1,610 | 29.7 (13.0) | 28.2 [19.5–38.4] | 26.20% | 371.7 (117.2) | 357.1 [288.5–446.3] | 5.50% | 20553 (6002) | 19677 [16477–23366] | 47.70% |
| BMI Tertile | T1 (Low) | 3,608 | 29.1 (13.1) | 27.6 [18.8–37.7] | 28.00% | 352.1 (111.1) | 342.6 [271.2–420.6] | 6.90% | 19832 (5455) | 19134 [16071–22755] | 42.80% |
| BMI Tertile | T2 (Medium) | 3,611 | 28.1 (12.5) | 26.8 [18.6–36.0] | 29.80% | 365.5 (112.6) | 356.9 [287.0–433.0] | 5.50% | 19912 (5503) | 19079 [16047–22955] | 42.90% |
| BMI Tertile | T3 (High) | 3,605 | 26.9 (12.0) | 25.1 [17.8–34.7] | 32.60% | 379.6 (118.7) | 367.0 [296.4–452.2] | 4.10% | 19443 (5387) | 18709 [15760–22169] | 39.80% |
| District | Urban core | 5,989 | 28.2 (12.9) | 26.6 [18.2–36.6] | 30.00% | 365.4 (113.1) | 354.4 [284.4–435.8] | 5.30% | 19766 (5422) | 19022 [16021–22661] | 42.20% |
| District | Outer district | 4,835 | 27.8 (12.2) | 26.4 [18.6–35.7] | 30.30% | 366.2 (116.7) | 356.2 [286.1–436.1] | 5.80% | 19683 (5489) | 18919 [15875–22542] | 41.30% |
| Temperature | T1 (Low) | 3,619 | 25.3 (12.2) | 23.2 [15.7–33.0] | 39.80% | 346.7 (113.3) | 337.5 [268.6–415.6] | 8.40% | 19378 (5470) | 18745 [15560–22279] | 39.90% |
| Temperature | T2 (Medium) | 3,650 | 28.3 (12.6) | 26.5 [18.8–36.4] | 29.30% | 370.9 (112.2) | 361.1 [289.3–440.6] | 4.30% | 20160 (5407) | 19472 [16450–23094] | 46.00% |
| Temperature | T3 (High) | 3,555 | 30.5 (12.4) | 29.3 [21.1–38.2] | 21.20% | 379.9 (116.1) | 368.9 [300.3–448.7] | 3.90% | 19644 (5451) | 18681 [15913–22302] | 39.50% |
| Humidity | T1 (Low) | 3,880 | 27.5 (12.8) | 25.8 [17.6–35.8] | 32.30% | 360.3 (114.0) | 348.9 [280.2–428.6] | 6.10% | 19814 (5667) | 19042 [15843–22835] | 43.10% |
| Humidity | T2 (Medium) | 3,480 | 28.7 (12.4) | 27.3 [19.3–36.7] | 27.40% | 370.5 (117.1) | 360.0 [289.8–441.9] | 5.40% | 19216 (5104) | 18572 [15757–22004] | 37.80% |
| Humidity | T3 (High) | 3,464 | 28.0 (12.6) | 26.3 [18.4–36.0] | 30.50% | 367.1 (112.8) | 357.8 [287.0–437.5] | 5.10% | 20149 (5504) | 19305 [16397–23005] | 44.40% |
| Precipitation | T1 (Low) | 7,360 | 27.9 (12.6) | 26.3 [18.2–35.9] | 30.60% | 365.0 (116.4) | 353.9 [284.1–435.8] | 5.80% | 19545 (5502) | 18805 [15752–22432] | 40.60% |
| Precipitation | T2 (Medium) | 3,464 | 28.4 (12.6) | 27.1 [18.8–36.5] | 29.30% | 367.4 (111.0) | 357.8 [287.7–436.3] | 4.90% | 20121 (5323) | 19279 [16469–22969] | 44.50% |
| Wind Speed | T1 (Low) | 3,702 | 27.5 (12.7) | 25.6 [17.6–35.9] | 32.60% | 366.5 (116.6) | 357.4 [282.2–438.7] | 5.60% | 19205 (5387) | 18540 [15456–22016] | 38.80% |
| Wind Speed | T2 (Medium) | 3,638 | 28.1 (12.6) | 26.4 [18.6–36.0] | 30.00% | 361.9 (111.3) | 350.8 [284.5–431.6] | 5.70% | 20144 (5444) | 19325 [16294–23062] | 44.40% |
| Wind Speed | T3 (High) | 3,484 | 28.6 (12.4) | 27.3 [19.1–36.5] | 27.80% | 369.0 (116.1) | 356.5 [289.4–439.1] | 5.20% | 19853 (5485) | 19042 [16115–22715] | 42.30% |
| Note: Data presented as mean ± SD, median [IQR], and proportion meeting deficiency (vitamin D <20 ng/mL; vitamin A <200 ng/mL) or excess (vitamin E ≥20,000 ng/mL) criteria. BMI tertiles: T1 (17.6–25.4), T2 (25.5–28.0), T3 (28.1–59.5 kg/m²). Meteorological variables stratified into tertiles except precipitation (median split). VD = vitamin D; VA = vitamin A; VE = vitamin E; IQR = interquartile range; BMI = body mass index. | | | | | | | | | | | |
|  |  |  |  |  |  |  |  |  |  |  |  |
|  |  |  |  |  |  |  |  |  |  |  |  |

| **Supplementary Table S2. Statistical Comparison of Vitamin Levels Across Stratification Factors** |  |  |  |
| --- | --- | --- | --- |
| **Stratification** | **Vitamin** | **Test** | ***P*** value |
| Season | Vitamin D | Kruskal-Wallis | <0.001 |
| Season | Vitamin A | Kruskal-Wallis | <0.001 |
| Season | Vitamin E | Kruskal-Wallis | <0.001 |
| Age group | Vitamin D | Kruskal-Wallis | <0.001 |
| Age group | Vitamin A | Kruskal-Wallis | 0.089 |
| Age group | Vitamin E | Kruskal-Wallis | <0.001 |
| BMI tertile | Vitamin D | Kruskal-Wallis | <0.001 |
| BMI tertile | Vitamin A | Kruskal-Wallis | <0.001 |
| BMI tertile | Vitamin E | Kruskal-Wallis | <0.001 |
| District | Vitamin D | Kruskal-Wallis | 0.256 |
| District | Vitamin A | Kruskal-Wallis | 0.820 |
| District | Vitamin E | Kruskal-Wallis | 0.192 |
| Temperature | Vitamin D | Kruskal-Wallis | <0.001 |
| Temperature | Vitamin A | Kruskal-Wallis | <0.001 |
| Temperature | Vitamin E | Kruskal-Wallis | <0.001 |
| Humidity | Vitamin D | Kruskal-Wallis | <0.001 |
| Humidity | Vitamin A | Kruskal-Wallis | <0.001 |
| Humidity | Vitamin E | Kruskal-Wallis | <0.001 |
| Precipitation | Vitamin D | Kruskal-Wallis | 0.019 |
| Precipitation | Vitamin A | Kruskal-Wallis | 0.141 |
| Precipitation | Vitamin E | Kruskal-Wallis | <0.001 |
| Wind speed | Vitamin D | Kruskal-Wallis | <0.001 |
| Wind speed | Vitamin A | Kruskal-Wallis | 0.054 |
| Wind speed | Vitamin E | Kruskal-Wallis | <0.001 |

| Supplementary Table S3. Logistic regression analyses of meteorological determinants and maternal factors associated with vitamin deficiencies and excess | | | | | |
| --- | --- | --- | --- | --- | --- |
| **Outcome** | **Predictor/Variable** | **Univariate OR (95% CI)** | ***P* value** | **Multivariable aOR (95% CI)** | ***P* value** |
| Vitamin D deficiency (<20 ng/mL) | Temperature (per 1°C) | 0.962 (0.957–0.966) | <0.001 | 0.970 (0.962–0.979) | <0.001 |
|  | Humidity (per 1%) | 0.996 (0.992–0.999) | 0.005 | 0.997 (0.994–1.001) | 0.158 |
|  | Precipitation (per 1 mm) | 0.996 (0.992–1.000) | 0.052 | 0.998 (0.993–1.003) | 0.409 |
|  | Wind speed (per 1 m/s) | 0.978 (0.965–0.991) | 0.001 | 1.001 (0.987–1.014) | 0.935 |
|  | Age 25–29 vs <25 | NA | NA | 0.707 (0.562–0.891) | 0.003 |
|  | Age 30–34 vs <25 | NA | NA | 0.543 (0.432–0.684) | <0.001 |
|  | Age ≥35 vs <25 | NA | NA | 0.475 (0.370–0.609) | <0.001 |
|  | BMI T2 vs T1 | NA | NA | 1.086 (0.979–1.206) | 0.119 |
|  | BMI T3 vs T1 | NA | NA | 1.244 (1.122–1.379) | <0.001 |
|  | Season: Summer vs Spring | NA | NA | 0.724 (0.624–0.841) | <0.001 |
|  | Season: Autumn vs Spring | NA | NA | 0.721 (0.640–0.813) | <0.001 |
|  | Season: Winter vs Spring | NA | NA | 0.952 (0.805–1.126) | 0.566 |
|  | District: Outer vs Urban | NA | NA | 0.990 (0.910–1.078) | 0.820 |
| Vitamin A deficiency (<200 ng/mL) | Temperature (per 1°C) | 0.960 (0.952–0.968) | <0.001 | 0.976 (0.959–0.994) | 0.008 |
|  | Humidity (per 1%) | 0.991 (0.985–0.997) | 0.004 | 0.993 (0.987–1.000) | 0.053 |
|  | Precipitation (per 1 mm) | 0.996 (0.987–1.005) | 0.362 | 1.002 (0.992–1.012) | 0.625 |
|  | Wind speed (per 1 m/s) | 0.988 (0.962–1.015) | 0.375 | 1.009 (0.982–1.036) | 0.498 |
|  | Age 25–29 vs <25 | NA | NA | 0.930 (0.597–1.524) | 0.76 |
|  | Age 30–34 vs <25 | NA | NA | 0.923 (0.595–1.508) | 0.734 |
|  | Age ≥35 vs <25 | NA | NA | 0.956 (0.593–1.608) | 0.859 |
|  | BMI T2 vs T1 | NA | NA | 0.765 (0.630–0.928) | 0.007 |
|  | BMI T3 vs T1 | NA | NA | 0.557 (0.450–0.686) | <0.001 |
|  | Season: Summer vs Spring | NA | NA | 1.041 (0.755–1.437) | 0.808 |
|  | Season: Autumn vs Spring | NA | NA | 1.098 (0.850–1.421) | 0.474 |
|  | Season: Winter vs Spring | NA | NA | 1.680 (1.211–2.342) | 0.002 |
|  | District: Outer vs Urban | NA | NA | 1.144 (0.968–1.351) | 0.115 |
| Vitamin E excess (≥20,000 ng/mL) | Temperature (per 1°C) | 0.998 (0.994–1.002) | 0.369 | 0.989 (0.981–0.998) | 0.011 |
|  | Humidity (per 1%) | 1.001 (0.998–1.004) | 0.358 | 1.002 (0.999–1.006) | 0.149 |
|  | Precipitation (per 1 mm) | 0.998 (0.994–1.002) | 0.346 | 0.997 (0.992–1.001) | 0.135 |
|  | Wind speed (per 1 m/s) | 1.018 (1.005–1.030) | 0.005 | 1.024 (1.011–1.037) | <0.001 |
|  | Age 25–29 vs <25 | NA | NA | 1.571 (1.234–2.014) | <0.001 |
|  | Age 30–34 vs <25 | NA | NA | 1.876 (1.477–2.401) | <0.001 |
|  | Age ≥35 vs <25 | NA | NA | 2.311 (1.794–2.996) | <0.001 |
|  | BMI T2 vs T1 | NA | NA | 0.992 (0.903–1.090) | 0.873 |
|  | BMI T3 vs T1 | NA | NA | 0.863 (0.785–0.949) | 0.002 |
|  | Season: Summer vs Spring | NA | NA | 0.895 (0.782–1.025) | 0.109 |
|  | Season: Autumn vs Spring | NA | NA | 1.148 (1.028–1.282) | 0.014 |
|  | Season: Winter vs Spring | NA | NA | 0.714 (0.608–0.839) | <0.001 |
|  | District: Outer vs Urban | NA | NA | 0.984 (0.911–1.064) | 0.686 |
| Note: OR = odds ratio; aOR = adjusted odds ratio (adjusted for age, BMI, season, district, and all meteorological variables). CI = confidence interval. NA = not applicable (variable not included in univariate analysis). | | | | | |
|  |  |  |  |  |  |

| **Supplementary Table S4. Restricted cubic spline models for dose–response analysis** | | | |
| --- | --- | --- | --- |
| **Vitamin** | **Outcome** | ***P*_overall_** | ***P*_nonlinear_** |
| Vitamin A | Fetal distress | <0.001 | 0.093 |
| Vitamin A | Premature rupture of membranes | <0.001 | 0.182 |
| Vitamin A | Meconium-stained amniotic fluid | 0.014 | 0.012 |
| Vitamin A | Low birth weight | <0.001 | 0.015 |
| Vitamin A | Macrosomia | <0.001 | 0.666 |
| Vitamin D | Fetal distress | <0.001 | 0.332 |
| Vitamin D | Premature rupture of membranes | 0.012 | 0.071 |
| Vitamin D | Meconium-stained amniotic fluid | 0.144 | 0.411 |
| Vitamin D | Low birth weight | 0.343 | 0.213 |
| Vitamin D | Macrosomia | <0.001 | 0.023 |
| Vitamin E | Fetal distress | 0.011 | 0.091 |
| Vitamin E | Premature rupture of membranes | 0.205 | 0.238 |
| Vitamin E | Meconium-stained amniotic fluid | 0.294 | 0.224 |
| Vitamin E | Low birth weight | 0.223 | 0.304 |
| Vitamin E | Macrosomia | <0.001 | 0.02 |
| Note: Poverall tests the overall significance of the association between maternal vitamin concentration and each outcome across the full exposure range, while Pnonlinear assesses departure from linearity. Models were fully adjusted for maternal age, pre-pregnancy body mass index, season, residential district, and gestational age at blood collection. Statistically significant P(nonlinear) (<0.05) indicates a nonlinear (e.g., U-shaped or threshold-like) relationship between vitamin levels and outcome risk.   | | | |
|  |  |  |  |
|  |  |  |  |
|  |  |  |  |
